# Association between maternal pre-pregnancy body mass index and offspring’s outcomes at 9 to 15 years of age

**DOI:** 10.1007/s00404-023-07184-5

**Published:** 2023-09-09

**Authors:** Alexander Lichtwald, Cathérine Weiss, Anja Lange, Till Ittermann, Heike Allenberg, Hans J. Grabe, Matthias Heckmann

**Affiliations:** 1 Department of Neonatology and Pediatric Intensive Care, University Medicine Greifswald, Greifswald, Germany; 2 Institute of Community Medicine, Division of Health Care Epidemiology and Community Health, University Medicine Greifswald, Greifswald, Germany; 3 Department of Psychiatry and Psychotherapy, University Medicine Greifswald, Greifswald, Germany

**Keywords:** Maternal pre-pregnancy underweight, Maternal pre-pregnancy overweight and obesity, Offspring’s social behavior, Offspring’s health, Offspring’s development

## Abstract

**Objective:**

Maternal pre-pregnancy underweight, overweight and obesity might increase the risk for worse short- and long-term outcome in the offspring. There is a need for further study into the relationship between maternal pre-pregnancy body mass index (BMI) and the combined outcome of physical development, state of health and social behavior in children. Question: Is maternal pre-pregnancy BMI associated with the child outcome in terms of physical development, state of health and social behavior (school and leisure time behavior) at the age of 9 to 15 years?

**Methods:**

In the population-based birth cohort study Survey of Neonates in Pomerania (SNIP) children at the age 9–15 years and their families were re-examined by questionnaire-based follow-up. 5725 mother–child pairs were invited to SNiP-follow-up. This analysis is based on the recall fraction of 24.1% (n = 1379). Based on the maternal pre-pregnancy BMI (ppBMI), 4 groups were formed: underweight (ppBMI < 19 kg/m^2^, n = 117), normal weight (ppBMI 19–24.99 kg/m^2^, n = 913, reference), overweight (ppBMI 25–30 kg). /m^2^, n = 237) and obesity (ppBMI > 30 kg/m^2^, n = 109).

**Results:**

In the multiple regression model, the BMI-z-score for children of mothers in the underweight group was −0.50 lower, and 0.50/1.07 higher in the overweight/obese group (p < 0.001) compared to reference at median age of 12 years. No differences were found in children of underweight mothers with regard to social behavior (interaction with friends and family), school and sports performance (coded from “very good” to “poor”), other leisure activities (watching television, using mobile phones, gaming), and health (occurrence of illnesses) compared to children of normal weight mothers. In contrast, maternal pre-pregnancy overweight and obesity were associated with lower school and sports performance, and higher screen time (smart phone, gaming, television) compared to children of normal weight mothers.

**Conclusion:**

Maternal pre-pregnancy overweight and obesity but not underweight was negatively associated with school performance and leisure time behavior in the offspring at 9–15 years of age.

**Supplementary Information:**

The online version contains supplementary material available at 10.1007/s00404-023-07184-5.

## What does this study add to the clinical work

**Table:** 

Pre-pregnancy maternal overweight and obesity is a negative risk factor for the offspring’s school performance and lei-sure time behavior. The incidence of non-communicable diseases is also high in this area, therefore prevention programs need to start before pregnancy.	

## Introduction

Maternal pre-pregnancy underweight, overweight and obesity might increase the risk for worse short- and long-term outcome in the offspring.

Maternal obesity is associated with an increased risk of gestational diabetes, preeclampsia, caesarean section, and miscarriage [1, 2]. Newborns born to obese mothers are at increased risk fetal macrosomia, congenital anomalies and admission to a neonatal unit [1, 3]. Longer-term consequences include an increased risk of obesity and metabolic syndrome in childhood and a lower intelligence quotient [1, 4, 5].

Maternal underweight also affects short-term perinatal outcomes and also health status in later life. Infants of underweight women are more exposed to higher risk of preterm birth, and to be small for gestational age (SGA) [6, 7]. Preterm birth may affect morbidity and mortality in adulthood [8, 9]. SGA and low birth weight are associated with cardiovascular disease in later life [10]. Furthermore, increasing odds ratios for psychiatric diagnoses were found with decreasing birth weight across the birth weight range [11]. Maternal nutritional status affects mental health of the offspring as inadequate gestational weight gain (GWG) was associated with an increased risk for nonaffective psychosis in offspring [12]. Smoking during pregnancy is one of the major risk factors for low birth weight and fetal growth restriction [13].

The prevalence of maternal pre-pregnancy overweight and obesity is high in industrialized countries, i.e. ranging from 29.2% to 63.0% in metropolitan cities in the United States [14]. In Germany, 40% of expecting mothers had a pre-pregnancy BMI > 25 kg/m^2^ in 2020 [15]. The prevalence of low pre-pregnancy BMI is also a significant amount in developed countries ranging from 4 to 12% depending on the used BMI classification and study [16–19]. In Germany, 11.7% of expecting mothers had a pre-pregnancy BMI < 20 kg/m^2^ in 2020 [15]. In the northeast of Germany, the population-based birth cohort study Survey of Neonates in Pomerania (SNiP) reported the prevalence of maternal pre-pregnancy BMI < 19 kg/m^2^, BMI > 25 kg/m^2^, and BMI > 30 kg/m^2^ was 10.7, 17.9, and 9.7%, respectively [2].

At the same time, the population project Study of Health in Pomerania (SHIP) investigates to what extent the high mortality in the northeastern adult German population can be explained by the risk-factor profile in that part of the country [20]. The SHIP investigators found a pronounced cardiometabolic risk factor and disease burden in the region. The following risk factors and diseases were higher than in any other part of Germany and occupied alarmingly high positions in international comparisons: alcohol consumption, obesity, metabolic syndrome, diabetes mellitus, arterial hypertension, and gallstone disease [21]. Furthermore, SHIP reported a rising prevalence of obesity and diabetes mellitus over a decade [21]. Birth cohorts like SNiP are needed to identify and monitor early antecedents of adult health to enable the development of preventive measures that specifically target these non-communicable diseases.

The aim of this analysis was to investigate the association between maternal pre-pregnancy BMI and child outcomes in terms of health, developmental and social aspects at the age of 9 to 15 years based on the follow-up of the SNiP [22].

## Materials and methods

### Study design

The present analysis is based on the birth cohort study ‘Survey of Neonates in Pomerania’ (SNiP-I). The design of the SNiP-I study has been described in detail by Ebner et al. [23]. In brief, the SNiP-I study was conducted from February 2002 to November 2008 in the region of Pomerania in Northeastern Germany. SNiP is a population-based, representative study in Germany that is able to describe the health and living conditions of newborns and their families comprehensively. It can contribute to similar cohort studies since data are accessible by external researchers.

**Fig. 1:**
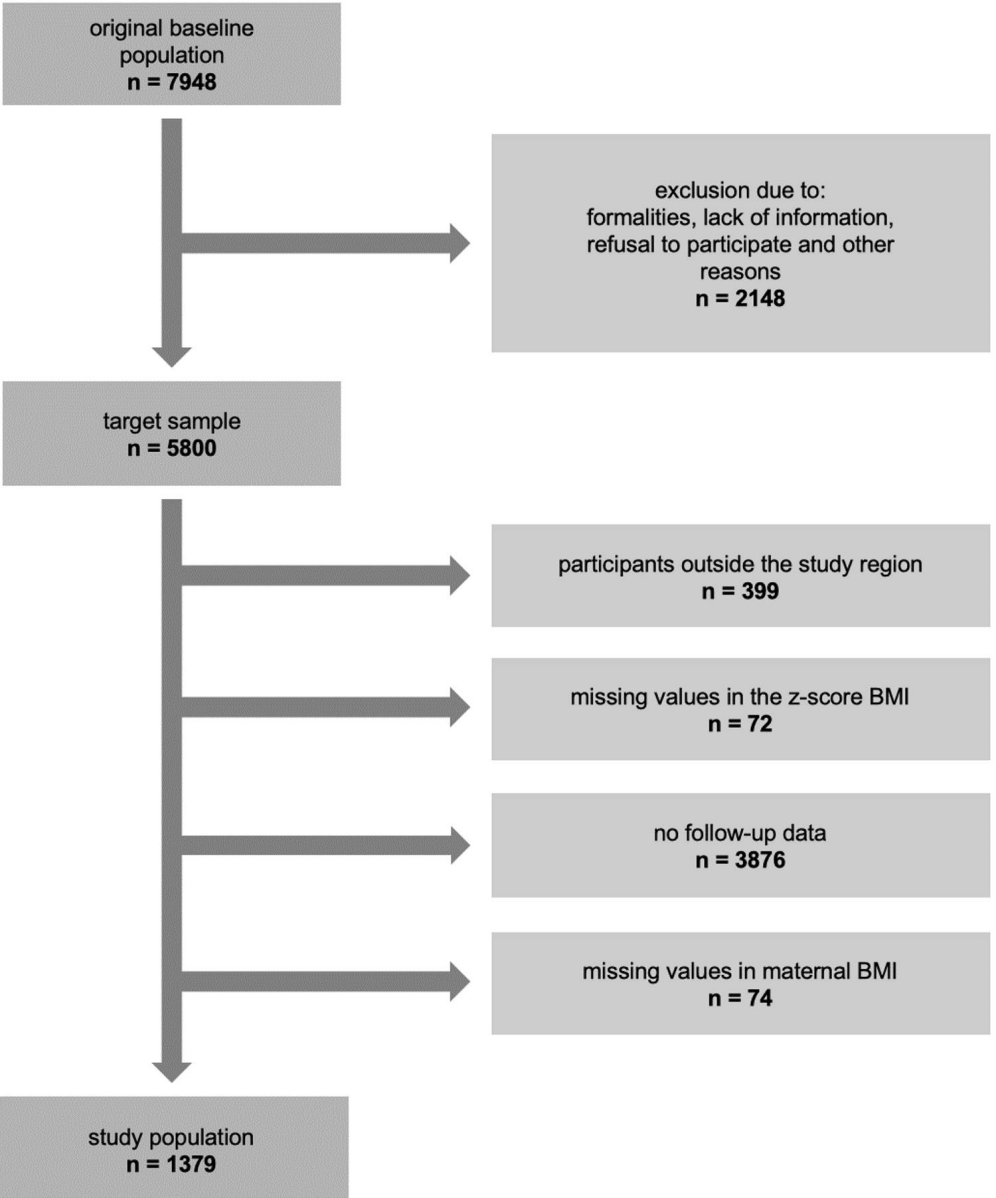
Flow chart of the selection process apply to data from the SNiP-I-cohort

All mothers from the SNiP-I birth cohort were recontacted when their children were from 9 to 15 years of age. The SNiP-I-Follow-up study was carried out between December 2016 and August 2017 which was described in detail by Kantorczyk et al. [22]. In brief, physical development, health status, and social behaviour (school and leisure behaviour) of children were analysed using a questionnaire comprising medical, epidemiological, and socio-economic data, associated health care risk factors, and life circumstances of newborns, children, and their parents. Out of 5725 children invited to participate in the SNiP-I-Follow-up study, 29% (n = 1665) children participated in the SNiP-I-Follow-up study, providing data on 1665 mothers-child dyads. Neither birthweight, nor sex nor the rate of preterm birth (< 37 weeks) differed significantly between participants of the follow-up and non-responders. Admission to neonatal care immediately after birth was slightly lower in responders. No differences were observed for mothers’ pre-pregnancy BMI or prevalence of gestational diabetes, but other maternal characteristics differed: mothers of participants in the SNiP-I-Follow-up study were older, had higher available monthly income and educational status, were less likely to smoke during pregnancy, and had more frequently declared their intentions to breast feed compared to mothers of non-responders [22]. Figure 1 illustrates the exclusion process to generate the sample for this analysis.

### Maternal variables

Height (cm) and pre-pregnancy body mass (kg) were reported by women using a standardised self-administrated questionnaire during the stay at the obstetrical ward [23]. For this analysis, women were categorized into four different BMI groups: “underweight” (< = 19 kg/m^2^), “normal weight” (19–24.99 kg/m^2^), “overweight” (25–29.99 kg/m^2^), and “obese” (> = 30 kg/m^2^). The definition of underweight for this study was set to <  = 19 kg/m^2^.

At Follow-Up, parents were asked to measure their own and actual child’s weight and height. These data were used further to calculate secondary variables, i.e. BMI (kg/m^2^), and percentiles for weight and height.

Gestational diabetes, pregnancy-induced arterial hypertension, and preeclampsia were included as pregnancy complications in the analyses.

The stratification pattern for educational level followed the already published pattern [2]. Persons without a school diploma, being still at school or with five years, or less, of secondary school, were pooled together and were referred to as having a low educational level. Persons with six years of secondary school (German ‘Realschulabschluss’) were included in the second level, referred to as the middle educational level. The third level included persons with eight years of secondary school (German ‘Fachhochschulreife’ or ‘Abitur’).

The need for housing space, electricity and other essentials does not increase proportionally with the higher number of members in the household. To account for this phenomenon, we have used equivalence scales, based on the OECD-modified scale [2].

Within our analyses, we did not quantify dose effects regarding alcohol and tobacco consumption. Only a dichotomous classification into “alcohol consumption/no alcohol consumption” and “smokers/non-smokers” was performed. All women who reported smoking at the current time were classified as smokers. Equivalently, those women with alcohol consumption were classified who currently consumed alcohol on a regular basis. The extent and duration of consumption of both stimulants were disregarded in both cases.

### Offspring variables

The following variables are addressed in the questionnaire to assess the child´s health status [22]: visual aids (If child uses any visual aids, and since when), hearing impairment (Does child suffer from any hearing losses), ADHD (Does child suffer from attention disorder and hyperactivity), headache (occurring of headache during last three months, type of applied therapy), allergies (Whether child suffers from any allergy or not, and if, what kind of allergy: hay fever, atopic eczema, allergic asthma, allergy to animal hairs, drugs or other substances), syncope (Whether child was unconscious within last 12 months or not), heart diseases (Whether child suffers from cardiac murmur, ventricular septal defect, atrial septal defect). Chronic diseases information was asked by a checklist, not official diagnoses according to ICD-10 system, for example coeliac disease, anorexia nervosa, diabetes mellitus, hypertension, epilepsy. Finally, Accident(s) within the last 12 months were recorded. Dental hygiene was assessed by frequency of daily dental hygiene, type toothpaste, frequency of visit by a dentist.

“Estimation of the child’'s well-being” includes the child’s well-being in terms of physical (Perception of physical well-being during last seven days, like filling sick, tired, or having power and endurance) and mental well-being (child’s self-esteem, child’s relations within the family, child’s friendship, and child’s perception of the school). The assessment was made by the child’s mother [22].

The variable “school” includes the type of school (e.g. elementary school, secondary school, high school, special school) that the child attends. In addition, it was recorded whether the child has skipped classes or had to repeat them [22].

The child’s leisure time activities are categorized as sports, outdoor and indoor activities. Within the variable sport, the school grade and the subjective parental evaluation of the child’s sporting achievements are taken into account. Outdoor activities include sports activities inside or outside a club and their regular frequency of practice. Indoor activities were defined as time spent watching TV, playing computer games, video games, other game consoles, or on a smartphone [22]. Interventionary studies involving animals or humans, and other studies that require ethical approval, must list the authority that provided approval and the corresponding ethical approval code.

### Potential mediators and confounder

**Fig. 2:**
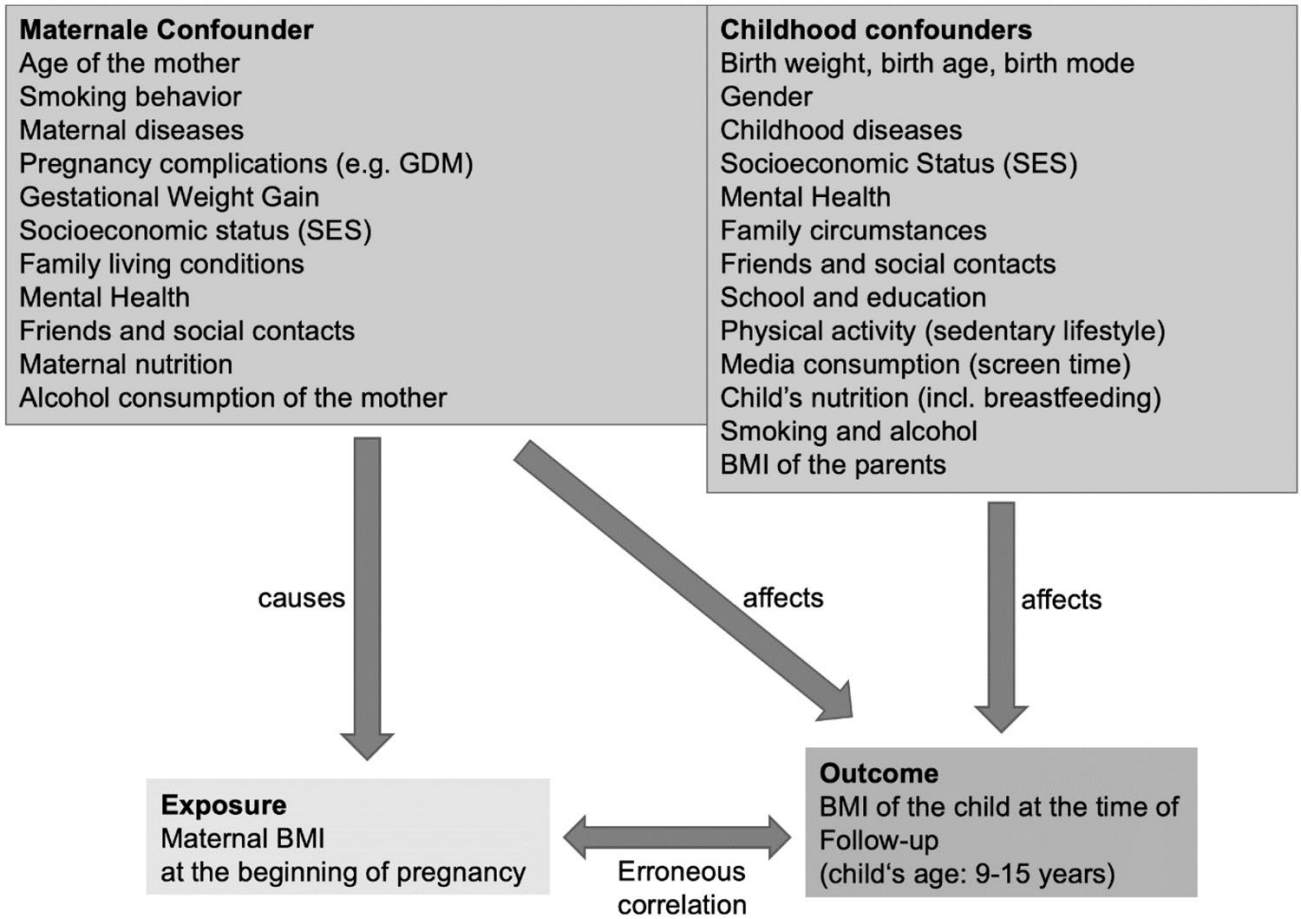
Influence of confounders on exposure and outcome

We identified the following variables as possible mediators with regard to the influence of maternal underweight on child outcome shown in Fig. 2 below:

It should be noted that the pre-pregnancy BMI is related to the offspring’s outcome. In addition, however, confounders must be taken into account that also have an influence, so that the BMI of the mother at the beginning of pregnancy is not the only direct causal factor for the child’s outcome. In the considered study age of the children from 9 to 15 years, especially child behaviors, such as media consumption or sports activities, influence the child outcome and their BMI. Ethnicity as a potential confounder could not be investigated in the study, as more than 98% of the study population had no migration background.

### Statistics

Statistical calculations were performed using STATA version 17.0 (StataCorp. LLC, USA). Data backup was performed using a Microsoft Access database (Microsoft Corporation, Redmond, WA, USA).

The mean (± standard deviation), median (including 25th and 75th percentiles), and number of subjects were obtained for continuous variables. Categorical data are reported as absolute numbers and percentages. For continuous and categorial data, Wilcoxon test and two-tailed x^2^ test were performed to compute p-values. Associations between maternal pBMI as independent variable and maternal and child outcomes as dependent variables were analysed using regression models adjusted for confounding. Confounders used are mentioned in each table. For continuous outcomes we used a linear regression model. Categorical data was analysed using multinomial logistic regression and for dichotomous outcomes we performed logistic regression. For the variables such as smartphone use, TV, gaming and school performance an ordered logistic regression was applied. Throughout, p values < 0.05 were considered statistically significant.

## Results

### Maternal characteristics

Of the 1665 mother-infant dyads in follow-up, pre-pregnancy BMI was available for 1379 mother-infant pairs.

Table 1 shows selected characteristics of mothers at baseline (childbirth) and follow-up. Mothers with low ppBMI were 2 years younger in median (p < 0.01) at baseline and 1 year younger at follow-up than mothers with normal BMI. Mothers with low ppBMI (13%) smoked nearly twice as often as mothers with normal pp weight (7.4%) at baseline. At follow-up, smoking was found three times higher (20.3%) of the mothers with normal pp weight compared to 25–27.5% in mothers in lower or higher ppBMI-classes. Monthly income was highest in normal pp weight mothers at follow-up compared to the other ppBMI-classes. Educational status was lower in mothers with pp obesity and pp compared to normal weight and underweight mothers.

**Table 1:** Maternal characteristics at baseline and follow-up stratified by pre-pregnancy body mass index (BMI) at baseline (childbirth) and at follow-up (9–15 years of age) of SNiP birth cohort

	N	Underweight (ppBMI < 19 kg/m^2^) n = 117	Normal weight (ppBMI 19–24.99 kg/m^2^) n = 913	Overweight (ppBMI 25–29.99 kg/m^2^) n = 237	Obese (ppBMI > / = 30 kg/m^2^) n = 109	p*
At baseline						
Pre-pregnancy BMI (kg/m^2^)	1379	18.4 (17.9; 18.7)	21.7 (20.6; 23.1)	26.7 (25.8; 28.0)	32.9 (31.1, 35.6)	< 0.005
Maternal age (years)	1376	27 (23; 30)	29 (26; 32)	29 (26; 33)	28 (24; 33)	0.001
Educational status	1351					< 0.005
< 10 years		9 (7.7)	46 (5.1)	25 (10.8)	15 (14.0)	
= 10 years		51 (44.0)	429 (47.8)	122 (52.8)	65 (60.8)	
> 10 years		29 (25.0)	236 (26.3)	51 (22.1)	15 (14.0)	
University		27 (23.3)	186 (20.7)	33 (14.3)	12 (11.2)	
Smoking during pregnancy	1344	15 (13.0)	66 (7.4)	25 (10.9)	11 (10.4)	0.09
Alcohol consumption during pregnancy	1359	29 (24.8)	233 (25.8)	54 (23.3)	16 (14.8)	0.09
Gestational diabetes	1379	1 (0.9)	33 (3.6)	19 (8.0)	14 (12.8)	< 0.005
Arterial hypertension	1379	8 (6.8)	26 (2.8)	19 (8.0)	10 (9.2)	< 0.005
Preeclampsia	1379	1 (1.7)	20 (2.2)	12 (5.1)	5 (4.6)	0.059
Mode of birth						0.076
Spontaneous	956	86 (73.5)	649 (71.16)	156 (65.82)	65 (60.19)	
Primary c-section	221	16 (13.68)	138 (15.13)	46 (19.41)	21 (19.44)	
Secondary c-section	146	10 (8.55)	93 (10.20)	30 (12.66)	13 (12.04)	
Operative	51	5 (4.27)	32 (3.51)	5 (2.11)	9 (8.33)	
At follow-up						
Maternal age (years)	1376	39 (35; 42)	40 (37; 45)	41 (38; 46)	39 (36; 45)	0.002
Smoking	1369	29 (25.0)	185 (20.3)	64 (27.5)	27 (25.0)	0.088
Equivalent income; €	989	1588 (1123; 2050)	1750 (1230; 2250)	1565 (1083; 2021)	1230 (780; 1750)	< 0.005
Employement status	1279					0.16
Unemployed		0 (0.0)	11 (1.3)	5 (2.4)	3 (3.1)	
Part-time		52 (46.0)	396 (44.9)	110 (52.4)	44 (45.4)	
Full-time		61 (54.0)	462 (53.8)	95 (45.2)	50 (51.5)	
Marital status	1370					0.048
Married		79 (68.1)	640 (70.4)	169 (71.9)	71 (65.1)	
Not married		32 (27.6)	223 (14.5)	51 (21.7)	31 (28.44)	
Divorced		4 (3.5)	46 (5.0)	11 (4.7)	6 (5.5)	
Widowed		1 (0.9)	1 (0.1)	4 (1.7)	1 (0.9)	

*ppBMI* pre-pregnancy BMI. Data are expressed as median, 25th or 75th percentile (continuous data) and as absolute numbers and percentages (categorical data)

*p-values derived from Wilcoxon tests (continuous data) and two-tailed x^2^ test (categorical data)

Supplemental Table 1 compares the sample analysed at follow-up (responders) with the baseline sample analysed by Domanski et al. with respect to maternal and neonatal parameters at baseline [7]. At follow-up, n = 76 mothers with pp underweight (median (quartiles) 18.4 (17.9; 18.7)) answered the questionnaire compared to n = 322 mothers with pp underweight (BMI 17.9 (17.3; 18.3)). Responders with pp underweight were in median 3 years older, smoked less during pregnancy (13 vs 39.8%), and had a higher socioeconomic status (in the following called “SES”) than the entire group of mothers with pp underweight at baseline. Responders with normal pp weight were also older, smoked less and had a higher SES than the entire group of mothers with normal pp weight at baseline.

### Offspring characteristics

At follow-up, there were no differences in offspring’s age, sex, but in weight and BMI (Table 2). Children of the low ppBMI group displayed a lower BMI and z-score BMI than the reference group whereas children of pp overweight and pp obese mothers showed a greater BMI. In addition, no differences in disease prevalence were detected between the groups. Low and normal ppBMI mothers reported a better performance in school and sports for their offspring than the other two groups.

**Table 2:** Offspring characteristics stratified by maternal pre-pregnancy weight at baseline (childbirth) and at follow-up (9–15 years of age) of SNiP birth cohort

	N	Underweight (ppBMI < 19 kg/m^2^) n = 117	Normal weight (ppBMI 19–24.99 kg/m^2^) n = 913	Overweight (ppBMI 25–29.99 kg/m^2^) n = 237	Obese (ppBMI > / = 30 kg/m^2^) n = 109	p*
At birth						
Gestational week of birth	1379	39 (38; 40)	39 (38; 40)	39 (38;40)	40 (38; 40)	0.014
Birth weight	1379					0.009
Small for GA	114	13 (11.1)	67 (7.3)	21 (8.9)	13 (11.9)	
Large for GA	151	4 (3.4)	96 (10.5)	37 (15.6)	14 (12.8)	
Admission to neonatal care	1375	20 (17.2)	149 (16.3)	44 (18.6)	23 (21.1)	0.578
At follow-up						
Age (years)	1379	12 (10; 14)	12 (10; 13)	12 (10; 13)	12 (10; 13)	0.07
Weight (kg)	1379	41 (32; 48)	40 (34; 51)	47 (36; 56)	50 (39; 60)	< 0.005
BMI (kg/m^2^)	1379	16.7 (14.2; 18.4)	17.6 (16.0; 20.0)	19.3 (16.7; 22.0)	20.5 (18.3; 23.5)	< 0.005
z-score BMI	1379	–0.6 (−1.4; 0.2)	0 (−0.8; 0.9)	0.6 (−0.4; 1.5)	1.2 (0.2; 2.0)	< 0.005
Sex	1379					0.82
Girl		52 (44.4)	422 (46.1)	114 (48.1)	54 (49.5)	
Boy		65 (55.6)	494 (53.9)	123 (51.9)	55 (50.4)	
Headache last 3 months	1366	58 (49.6)	409 (45.0)	118 (50.0)	47 (44.8)	0.695
Syncope	1367	14 (12.0)	57 (6.3)	17 (7.3)	6 (5.6)	0.13
Diabetes mellitus Typ 1	1344	1 (0.9)	5 (0.6)	1 (0.4)	1 (0.9)	0.93
Arterial hypertension	1335	0 (0.0)	7 (0.8)	3 (1.3)	2 (1.9)	0.43
Bronchitis (asthmatic, spastic, and/or obstructive)	1340	20 (17.7)	137 (15.4)	36 (15.7)	17 (15.7)	0.94
Pseudo-Krupp	1344	18 (15.7)	189 (21.2)	51 (22.4)	19 (17.6)	0.403
Allergy	1351	34 (29.6)	254 (28.4)	63 (27.2)	26 (24.1)	0.778
Eating disorder (not specified)	1338	3 (2.6)	13 (1.5)	2 (0.9)	2 (1.9)	0.64
Colitis ulcerosa	1326	0 (0.0)	3 (0.3)	3 (1.4)	0 (0.0)	0.16
School performance	1356					0.016
Above average		31 (26.5)	233 (25.9)	39 (16.7)	21 (19.8)	
Average		77 (65.8)	627 (69.7)	175 (74.8)	77 (72.6)	
Below average		6 (5.1)	31 (3.5)	18 (7.7)	7 (6.6)	
Insufficient		3 (2.6)	8 (0.9)	2 (0.9)	1 (0.9)	
Sports performance	1274					< 0.005
1 = very good		41 (39.0)	289 (33.9)	42 (19.4)	22 (22.0)	
2 = good		47 (44.8)	414 (48.6)	105 (48.4)	39 (39.0)	
3 = satisfactory		16 (15.2)	123 (14.4)	56 (25.8)	35 (35.0)	
4 = sufficient and less		1 (1.0)	26 (3.1)	14 (6.4)	4 (4.0)	
Outdoor activity	1355					0.29
Nearly every day		47 (41.6)	439 (48.7)	107 (45.7)	59 (55.7)	
3-5d/week		30 (26.6)	241 (26.7)	59 (25.2)	30 (28.3)	
1-2d/week		23 (20.4)	140 (15.5)	45 (19.2)	9 (8.5)	
< 1-2d/week		11 (9.7)	75 (8.3)	23 (9.8)	7 (6.6)	
Never		2 (1.8)	7 (0.8)	0 (0.0)	1 (0.9)	
Smartphone weekdays	1336					0.06
No use		36 (32.4)	321 (36.0)	84 (36.7)	24 (22.9)	
30 min		25 (22.5)	234 (26.3)	44 (19.2)	29 (27.6)	
1–2 h		30 (27.0)	244 (27.4)	74 (32.3)	36 (34.3)	
3–4 h		11 (9.9)	58 (6.5)	14 (6.1)	11 (10.5)	
> 4 h		9 (8.1)	34 (3.8)	13 (5.7)	5 (4.8)	
Smartphone weekends	1337					0.18
No use		29 (26.6)	393 (32.7)	76 (32.9)	22 (21.0)	
30 min		30 (18.4)	179 (20.0)	31 (13.4)	21 (20.0)	
1–2 h		29 (26.6)	209 (23.4)	66 (28.6)	28 (26.6)	
3–4 h		16 (14.7)	125 (14.0)	36 (15.6)	19 (18.1)	
> 4 h		15 (13.8)	87 (9.8)	22 (9.5)	15 (14.3)	
Gaming weekdays	1361					0.06
No use		56 (48.3)	378 (41.6)	93 (40.1)	36 (34.3)	
30 min		31 (26.7)	283 (31.2)	63 (27.2)	29 (27.6)	
1–2 h		24 (20.7)	223 (24.6)	70 (30.2)	36 (34.3)	
3–4 h		3 (2.6)	22 (2.4)	6 (2.6)	4 (3.8)	
> 4 h		2 (1.7)	2 (0.2)	0 (0.0)	0 (0.0)	
Gaming weekends	1343					0.008
No use		28 (25.2)	202 (22.4)	54 (23.7)	12 (11.5)	
30 min		36 (32.4)	227 (25.2)	44 (19.3)	24 (23.1)	
1–2 h		25 (22.5)	312 (34.7)	86 (37.7)	41 (39.4)	
3–4 h		16 (14.4)	126 (14.0)	30 (13.2)	24 (23.1)	
> 4 h		6 (5.4)	33 (3.7)	14 (6.1)	3 (2.9)	
TV weekdays	1359					< 0.005
No use		26 (22.8)	117 (12.9)	16 (6.9)	4 (3.8)	
30 min		31 (27.2)	295 (32.5)	56 (24.2)	20 (18.9)	
1–2 h		46 (40.4)	434 (47.8)	133 (57.6)	70 (66.0)	
3–4 h		7 (6.1)	59 (6.5)	23 (10.0)	10 (9.4)	
> 4 h		4 (3.5)	3 (0.3)	3 (1.3)	2 (1.9)	
TV weekends	1333					< 0.005
No use		2 (1.8)	30 (3.4)	1 (0.4)	0 (0.0)	
30 min		9 (8.3)	59 (6.6)	11(4.9)	5 (4.8)	
1–2 h		61 (56.0)	460 (51.5)	100 (44.1)	37 (35.6)	
3–4 h		26 (23.9)	382 (31.6)	86 (37.9)	46 (44.2)	
> 4 h		11 (10.1)	62 (6.9)	29 (12.8)	16 (15.4)	
Breastfeeding child	1337	96 (83.5)	730 (81.9)	162 (71.4)	64 (61.5)	< 0.005
Friendship harmony	1347					0.013
Often/always		109 (96.4)	848 (94.8)	205 (88.0)	99 (93.4)	
Sometimes		2 (1.8)	36 (4.0)	23 (9.9)	4 (3.8)	
Rarely/never		2 (1.8)	11 (1.2)	5 (2.1)	3 (2.8)	
Family harmony	1368					< 0.005
Often/always		96 (82.8)	826 (90.8)	211 (90.2)	95 (88.0)	
Sometimes		18 (15.5)	77 (8.5)	22 (9.4)	13 (12.0)	
Rarely/never		2 (1.7)	7 (0.7)	1 (0.6)	0 (0.0)	
Child comfort at home	1361					0.008
Often/always		105 (93.8)	885 (97.5)	228 (97.9)	103 (95.4)	
Sometimes		7 (6.2)	19 (2.1)	5 (2.2)	5 (4.6)	
Rarely/never		0 (0.0)	4 (0.4)	0 (0.0)	0 (0.0)	
Family dispute	1361					< 0.005
Rarely/never		98 (86.7)	847 (93.4)	207 (88.8)	92 (85.2)	
Sometimes		11 (9.7)	22 (9.4)	22 (9.4)	14 (13.0)	
Often/always		4 (3.5)	11 (1.2)	4 (1.7)	2 (1.8)	
Alcohol consumption	1367	7 (6.1)	58 (6.4)	11 (4.7)	8 (7.4)	0.75
Smoking	1367	1 (0.9)	5 (0.6)	2 (0.9)	4 (3.7)	0.011

*ppBMI*  pre-pregnancy BMI. Data are expressed as median, 25th or 75th percentile (continuous data) and as absolute numbers and percentages (categorical data)

*p-values derived from Wilcoxon tests (continuous data) and two-tailed x^2^ test (categorical data)

Indoor activities also differed between the four groups. Although the lowest total time spent watching TV and gaming was reported in the low ppBMI group, in terms of excessive device use, the proportion of children in the low ppBMI group is significantly higher than compared to the normal group.

The children's mental and their self-esteem was the same across all groups (data not shown). Mothers of the low ppBMI group reported a slightly more disrupted family harmony in comparison to the others, but the harmony of friendship was most stable in the low ppBMI group.

### Associations of maternal characteristics with pre-pregnancy BMI

Low ppBMI was associated with younger age with after adjustment for confounders but neither with maternal smoking in pregnancy nor at follow-up (Table 3). Maternal z-BMI at follow-up was associated with higher z-score BMI for the pre-pregnancy groups of overweight and obese mothers but no associations were observed in low ppBMI mothers. A maternal ppBMI > 25 kg/m^2^ was associated with a lower SES (education, income and unemployment).

**Table 3:** Association of maternal pre-pregnancy BMI with maternal socioeconomic status at follow-up of the SNiP birth cohort (child age 9–15 years)

Maternal characteristics	N	Underweight (ppBMI < 19 kg/m^2^) n = 117	Overweight (ppBMI 25–29.99 kg/m^2^) n = 237	Obese (ppBMI > / = 30 kg/m^2^) n = 109
		Relative risk ratio (95% confidence interval)		
Educational status°	1348			
< 10 years		0.97 (0.41; 2.27)	3.46 (1.89; 6.33)**	5.56 (2.45; 12.63)**
= 10 years		0.93 (0.57; 1.52)	1.31 (0.91; 1.90)	2.38 (1.33; 4.27)**
University		1.47 (0.83; 2.61)	0.72 (0.44; 1.17)	0.98 (0.44; 2.16)
Income per 1000 €°	819	0.91 (0.52;1.58)	0.54 (0.41; 0.71)**	0.27 (0.18; 0.40)**
Employment°	1260			
Unemployed		–	3.00 (0.79; 11.38)	7.49 (2.27; 24.62)**
Part-time		1.8 (1.01; 3.27)*	1.37 (1.05; 1.79)*	1.17 (0.85; 1.61)

Maternal age and educational status were analysed by multinomial logistic regression and smoking behavior by logistic regression. Values adjusted for the following variables: °maternal age; °° maternal age, educational status. Z-score BMI was analyzed using linear regression adjusted for maternal age, smoking, alcohol consumption and educational status

*pBMI*  pre-pregnancy BMI

*p-value < 0.05

** p-value < 0.005

### Associations of offspring outcomes with pre-pregnancy BMI

After adjustment for the maternal variables age, smoking, alcohol consumption and educational status, the offsprings z-score BMI stayed significantly associated to ppBMI (Table 4). Our results showed a −0.5 lower z-score BMI for children in the low ppBMI group than in the normal ppBMI group. Equivalently, higher child z-score BMI is associated with maternal overweight and obesity.

**Table 4:** Association of maternal pre-pregnancy BMI with offspring outcomes at follow-up of the SNiP birth cohort (child age 9–15 years)

Offspring outcome	N	Underweight (ppBMI < 19 kg/m^2^) n = 117	Overweight (ppBMI 25–29.99 kg/m^2^) n = 237	Obese (ppBMI > / = 30 kg/m^2^) n = 109
		β (95% confidence interval)		
z-score BMI°	1300	−0.50 (−0.74; −0.26)**	0.50 (0.31; 0.68)**	1.07 (0.81; 1.32)**
		Odds ratio (95% confidence interval)		
Smartphone°°				
Weekdays	1165	1.18 (0.80; 1.76)	1.09 (0.79; 1.49)	1.86 (1.22; 2.83)**
Weekend	1168	1.09 (0.74; 1.61	0.99 (0.73; 1.34)	1.75 (1.15; 2.67)*
Gaming°°				
Weekdays	1188	0.80 (0.54; 1.18)	1.11 (0.83; 1.50)	1.21 (0.79; 1.85)
Weekend	1175	0.77 (0.53; 1.12)	1.01 (0.76; 1.35)	1.59 (1.06; 2.38)*
TV°°				
Weekdays	1185	0.72 (0.48; 1.07)	1.64 (1.21; 2.22)**	1.70 (1.11; 2.60)*
Weekend	1162	0.87 (0.58; 1.30)	1.53 (1.13; 2.08)*	1.81 (1.18; 2.77)*
School performance°° (coded from very good to bad)	1184	1.02 (0.65; 1.59)	1.49 (1.04; 2.15)*	1.35 (0.80; 2.26)
Admission to a health center°	209	1.81 (0.56; 5.86)	0.86 (0.41; 1.79)	1.11 (0.54; 2.29)
Breastfeeding child°	1245	1.22 (0.52; 2.89)	0.77 (0.54; 1.09)	0.38 (0.26; 0.55)**
		Relative risk ratio (95% confidence interval)		
Family harmony°	1274			
Often/always		(Base outcome)	(Base outcome)	(Base outcome)
Sometimes		1.58 (0.67; 3.72)	1.38 (0.88; 2.18)	1.23 (0.72; 2.09)
Rarely/never		–	0.91 (0.18; 4.56)	0.70 (0.12; 4.20)
Child´s comfort at home°	1267			
Often/always		(Base outcome)	(Base outcome)	(Base outcome)
Sometimes		0.66 (0.08; 5.11)	0.86 (0.36; 2.03)	0.95 (0.37; 2.42)
Rarely/never		–	0.62 (0.05; 7.15)	0.61 (0.05; 7.36)
Family dispute°	1267			
Rarely/never		(Base outcome)	(Base outcome)	(Base outcome)
Sometimes		1.46 (0.49; 4.35)	1.84 (1.09; 3.12)*	1.93 (1.09; 3.46)*
Often/always		1.38 (0.16; 12.09)	1.36 (0.44; 4.13)	1.13 (0.32; 4.05)
Sports performance°	1185			
Very good		(Base outcome)	(Base outcome)	(Base outcome)
Below average		0.92 (0.37; 2.26)	1.44 (1.00; 2.07)	2.30 (1.54; 3.42)**
Insufficient		–	2.27 (0.97; 5.28)	3.48 (1.47; 8.23)*

Screentime (gaming, TV, smartphone) and school performance were analyzed by ordered logistic regression; admission to health care and breastfeeding by logistic regression; family harmony, child comfort at home and family dispute by multinomial logistic regression and the z-score BMI by linear regression

*ppBMI*  pre-pregnancy BMI

*p-value < 0.05

**p-value < 0.005

°Values adjusted for the variables maternal age, maternal smoking, alcohol consumption and maternal educational status

°°Adjusted for maternal age, maternal smoking, participation in a sport club, maternal educational status, maternal marital status, maternal employment status, child’s age at the follow-up

Adjusted logistic regression revealed no association between low BMI and children's indoor activities, school performance, or well-being. On the other hand, the odds for a more extensive use of smartphone usage, gaming and TV time were significantly associated with with overweight and obese ppBMI (Table 4).

Moreover, children of overweight mothers had a higher odds ratio for worse school grades. In addition, the risk for having a worse grade in school sports than the reference group is increased in the obesity group but not the other two.

Table 5 discriminates maternal variables for offspring outcomes. To investigate the discriminative ability of maternal variables on the offspring outcomes, we report the R^2^ (for z-score BMI) and area under the curves (for selected dichotomous outcomes) based on individual probabilities derived from bivariable logistic regression models.

**Table 5:** Discrimination of maternal variables for offspring outcomes in the SNiP birth cohort (child age 9–15 years)

	Follow-up data available					
	Pre-pregnancy BMI	Maternal age	education	(Equivalent income)	Employment	Smoking during pregnancy
	R^2^					
z-score BMI	0.094	0.003	0.032	0.018	0.000	0.004
Area under the curve						
Smartphone						
Weekdays	0.501	0.595	0.652	0.637	0.532	0.554
Weekend	0.505	0.591	0.611	0.608	0.544	0.533
Gaming						
Weekdays	0.515	0.559	0.653	0.640	0.543	0.535
Weekend	0.543	0.528	0.606	0.591	0.533	0.517
TV*						
Weekdays	0.547	0.598	0.693	0.662	0.598	0.540
Weekend	0.580	0.577	0.652	0.594	0.529	0.525
Sports performance**	0.605	0.528	0.608	0.637	0.591	0.533

R^2^ based on linear regression model; the area under the curve was calculate from the individual probabilities taken from logistic regression

*Dichotomized at > 2 h versus <  = 2 h

**Dichotomized at sufficient and less or satisfactory versus good or very good

## Discussion

This analysis investigated the association between maternal ppBMI and child outcomes at the age of 9 to 15 years in terms of health, developmental and social aspects based on the follow-up of the SNiP [22].

In the SNiP chohort, maternal ppBMI was associated with child’s BMI at follow-up. A lower z-score BMI was found in the offspring of mothers with pp underweight whereas a higher z-score BMI was found in children of mothers with pp overweight or obesity compared to those with normal pp weight. This is in accordance with recent literature [24, 25]. Maternal pp overweight and obesity are important risk factors for childhood obesity among other early predictors like smoking during pregnancy, gestational weight gain or infant sleep patterns [25, 26].

In SNiP, maternal pp overweight was associated with lower school performance and maternal pp obesity with lower sport performance in the offspring after correcting for SES. The Millennium Cohort Study from the United Kingdom showed that maternal ppBMI was negatively associated with children’s cognitive performance at age 5 and age 7 [27]. The United States Collaborative Perinatal Project reported that maternal ppBMI displayed an inverted U-shaped associations with child IQ [5]. Maternal pre-pregnancy overweight/obesity (ppBMI ≥ 25 kg/m^2^) with extremely excessive gestational weight gain were associated with increased offspring’s intellectual developmental disorders in a large Swedish cohort study [28]. This association was also found in a Chinese birth cohort study when children’s cognitive development was tested [29].

High media usage in children is related to poorer cognition, language, and social–emotional skills. In SNiP-follow-up, the odds for extensive screen time including television, pc gaming and mobile phone activity were higher in children born to mothers with pp overweight and/or pp obesity. It was shown that greater levels of screen time are associated with poorer physical health and obesity in later life [30]. At SNiP-follow-up, we did not find differences in child’s health status with respect to maternal ppBMI. In contrast, a recent meta-analysis who reported that children whose mothers were obese at the beginning of pregnancy have an increased risk of developing asthma or wheezing episodes between the ages of 14 months and 16 years [31]. Another study which was not part of the meta-analysis confirmed this result [32].

In SNiP, a high incidence of headache was reported in half of the children regardless of maternal ppBMI. This is in line with the literature [33]. Higher screen time is associated with lower psychological well-being and mental health issues, and particularly with the incidence of headache in school-aged children and adolescents [34–36].

Results from a large meta-analysis demonstrate an association between maternal prenatal smoking and childhood overweight [37]. At SNiP-follow-up, mothers smoked twice as often compared to pregnancy without a significant association to maternal ppBMI. However, it is known that significant proportion of women who had quit smoking during pregnancy, relapsed postpartum [38, 39]. In our analysis, the incidence of smoking during pregnancy among non-responders to follow-up was twice as high as among responders and particularly three times higher in the entire group of pp underweight compared to those who respond [7]. This is a substantial bias of our follow-up study.

Breast feeding is an important preventive measure against later metabolic syndrome and obesity [40]. In SNiP-follow-up, breast-feeding rates were 10/20% lower in the pp overweight/ pp obesity group compared to reference or pp underweight.

In SNiP, the prevalence of maternal pp underweight (ppBMI < 19 kg/m^2^) was 8.5% and the prevalence of maternal ppBMI > 25 was 25.1% at baseline 2002–2008 which displays in the range reported by others [16–18]. More recent data from Germany indicate that the ppBMI > 25 was 40% in expecting women in 2020 [15].

At SNiP-follow-up, mothers with pp underweight were in median one year younger compared to reference which goes in line with the baseline cohort. However, non-responder to follow-up were in median three years younger than responder which may create bias. In our baseline analysis, pp underweight was associated with younger age and smoking in pregnancy [7].

The association between maternal ppBMI and the offspring variables may be jointly mediated by lifestyle, SES, and genes which cannot be accounted for in this investigation. SES is related to physical and psychosocial health of children and adolescents [41]. Studies have shown particularly an association between a low SES and childhood overweight and obesity as well as a poorer physical health in later life [42–46]. In SNiP-follow-up, maternal overweight and obesity was associated with a lower SES, presented by a lower income, lower education and higher risk for part-time jobs or unemployment.

Further discrimination of maternal variables for offspring outcomes in SNiP by linear and logistic regression shows that the R^2^ is best for ppBMI for the offspring z-score BMI, and education plays the greatest role in the other selected outcomes (Table 5).

In SNiP, family harmony and child’s comfort at home was not associated with maternal ppBMI although children of mothers with pp obesity had greater odds of dispute with their parents. Family instability influences the cognitive and educational outcomes and may potentiate the effect of maternal ppBMI on offspring’s outcome [47–49].

### Strengths and limitations

The strengths of our analysis are the high population coverage of SNiP-I at baseline, the large number of participants, homogeneous ethnic compositions, and a geographically defined study region. Moreover, the collection of population-based data in rural, sparsely populated areas of Germany is a rarity. A comprehensive dataset including physical development, health status, and social behaviour (school and leisure behaviour) of children were available together with medical, epidemiological, and socio-economic data of the family at follow-up [22]. SNiP is geographically linked to the Study of Health in Pomerania (SHIP), which is one of Europe’s most representative and comprehensive prospective cohort studies of adult health [20].

A major limitation is that the sample was shifted to higher levels of SES, less mothers with low or high ppBMI, and less mothers smoking during pregnancy (suppl. table 1). Therefore, in contrast to baseline, the follow-up is no longer representative for the catchment area of SNiP. The comparisons of responders and non-responders confirmed the general bias towards participants with higher SES observed in other national or international studies [50, 51].

Large-scale genetic studies have identified genetic variants affecting body mass index in adults and weight gain in children [52]. However, genetic analyses were not available in our cohort.

## Conclusions

Maternal pp overweight and obesity but not underweight was negatively associated with school performance and leisure time behavior in the offspring at 9–15 years of age. Maternal pp overweight and obesity was associated with modifiable risk factors like less breast feeding, sedentary behaviors, more screen time, and low SES. Due to the high incidence of non-communicable disease in adults in the same area, prevention programs should start as early as possible, i.e. before pregnancy, with respect to control ppBMI, breast feeding promotion, smoking cessation and reducing screen-time.

### Supplementary Information

Below is the link to the electronic supplementary material.

Supplementary file1 (DOCX 29 KB)

## Data Availability

The paper is based on the data collected during the study ‘Survey of Neonates in Pomerania’ between 2002 and 2008 and ‘Survey of Neonates in Pomerania-I-Follow-up study’ between 2016 and 2017. Both are conducted at the University Medicine Greifswald, Greifswald, Germany. Data from SNIP are available via https://www.medizin.unigreifswald.de/kind_med/index.php?id=759. The repository is managed by the Research Cooperation Community Medicine (RCC) of the University of Greifswald, Germany. This data repository allows access to any researcher who has previously registered and requested access. In addition to online application tools for data access, it also provides a data dictionary. The RCC decides whether and to what extent to grant access to the data based on scientific guidelines after the user’s request.
