# Supplementary material for: Association between maternal pre-pregnancy body mass index and offspring’s outcomes at 9 to 15 years of age
Source: Arch Gynecol Obstet. 2023 Sep 9;309(1):105–18. doi: 10.1007/s00404-023-07184-5 (PMC10770235; doi:10.1007/s00404-023-07184-5)
Supplement: Supplementary file 1 — Supplementary file1 (DOCX 29 KB) [file 404_2023_7184_MOESM1_ESM.docx]

Original Article – Maternal Fetal Medicine
Association between maternal pre-pregnancy body mass index and offspring’s outcomes at 9 to 15 years of age

Alexander Lichtwald^1^*, Cathérine Weiss^1^*, Anja Lange^1^, Till Ittermann^2^, Heike Allenberg^1^, Hans J. Grabe^3^, Matthias Heckmann^1+^

^1^ Department of Neonatology and Pediatric Intensive Care, University Medicine Greifswald, Greifswald, Germany

^2^ Institute of Community Medicine, Div. of Health Care Epidemiology and Community Health, University Medicine Greifswald, Germany

^3^ Department of Psychiatry and Psychotherapy, University Medicine Greifswald, Greifswald, Germany

***** Authors contributed equally. Correspondence: AL. alexander.lichtwald@med.uni-greifswald.de; Tel.: +49-3834-866421. CW. s-caschw@uni-greifswald.de; Tel.: +49-3834-866421.

**^+^** Orchid-ID M.H.:0000-0002-5260-264X

**Supplemental Table 1.** Baseline maternal characteristics of the study population stratified by response at follow-up of the SNiP birth cohort (child age 9-15 years)

|  | **Follow-up data available** | | **p*** |
| --- | --- | --- | --- |
|  | **Yes**  (n=1524) | **No**  (n=3866) |  |
| **Maternal characteristics** |  |  |  |
| Gestational week of birth; weeks | 39 (38; 40) | 39 (38; 40) | 0.650 |
| Maternal age; years | 29 (25; 32) | 26 (23; 31) | <0.001 |
| Pre-pregnancy BMI; kg/m^2^  BMI < 19  19 ≤ BMI < 25  BMI ≥ 25 | 22.3 (20.6; 24.9)  8.9%  66.5%  24.6% | 22.5 (20.3; 25.6)  11.3%  60.6%  28.1% | 0.483  <0.001 |
| Educational status  < 10 years  = 10 years  > 10 years  University | 7.0%  49.6%  24.5%  18.9% | 18.9%  53.7%  18.3%  9.1% | <0.001 |
| Equivalent income; € | 1326 (796; 1679) | 866 (505; 1371) | <0.001 |
| Employment status  Full-time  Part-time  Unemployed | 55.5%  22.1%  22.5% | 45.2%  24.2%  30.6% | <0.001 |
| Smoking during pregnancy | 9.1% | 23.7% | <0.001 |
| Alcohol consumption during pregnancy | 24.2% | 21.8% | 0.070 |
| Parity  First birth  Second birth  Third birth  Four + birth | 46.7%  31.9%  13.3%  8.2% | 46.0%  29.1%  13.1%  11.9% | 0.001 |

Data are expressed as median, 25^th^ and 75^th^ percentile for continuous variables or as percentage for
categorical data
*Mann-Whitney-U test for continuous data and χ^2^ test for categorical data
